# Evaluation of Hyperparameter Optimization in Machine and Deep Learning Methods for Decoding Imagined Speech EEG

**DOI:** 10.3390/s20164629

**Published:** 2020-08-17

**Authors:** Ciaran Cooney, Attila Korik, Raffaella Folli, Damien Coyle

**Affiliations:** 1 Intelligent Systems Research Centre, Ulster University, Londonderry BT48 7JL, UK; korik-a@ulster.ac.uk (A.K.); dh.coyle@ulster.ac.uk (D.C.); 2 Institute for Research in Social Sciences, Ulster University, Jordanstown BT37 0QB, UK; r.folli@ulster.ac.uk

**Keywords:** electroencephalography (EEG), brain–computer interface (BCI), convolutional neural networks (CNN), deep learning, machine learning, hyperparameter optimization, imagined speech

## Abstract

Classification of electroencephalography (EEG) signals corresponding to imagined speech production is important for the development of a direct-speech brain–computer interface (DS-BCI). Deep learning (DL) has been utilized with great success across several domains. However, it remains an open question whether DL methods provide significant advances over traditional machine learning (ML) approaches for classification of imagined speech. Furthermore, hyperparameter (HP) optimization has been neglected in DL-EEG studies, resulting in the significance of its effects remaining uncertain. In this study, we aim to improve classification of imagined speech EEG by employing DL methods while also statistically evaluating the impact of HP optimization on classifier performance. We trained three distinct convolutional neural networks (CNN) on imagined speech EEG using a nested cross-validation approach to HP optimization. Each of the CNNs evaluated was designed specifically for EEG decoding. An imagined speech EEG dataset consisting of both words and vowels facilitated training on both sets independently. CNN results were compared with three benchmark ML methods: Support Vector Machine, Random Forest and regularized Linear Discriminant Analysis. Intra- and inter-subject methods of HP optimization were tested and the effects of HPs statistically analyzed. Accuracies obtained by the CNNs were significantly greater than the benchmark methods when trained on both datasets (words: 24.97%, *p* < 1 × 10^–7^, chance: 16.67%; vowels: 30.00%, *p* < 1 × 10^–7^, chance: 20%). The effects of varying HP values, and interactions between HPs and the CNNs were both statistically significant. The results of HP optimization demonstrate how critical it is for training CNNs to decode imagined speech.

## 1. Introduction

Brain–computer interfaces (BCIs) are a movement-independent communicative device, enabling users to interact with a computer through brain activity alone [1]. Traditional approaches to BCIs have harnessed brain activity corresponding to mental states such as motor imagery (MI) (e.g., [2]) or steady state visually-evoked potential (SSVEP) (e.g., [3]) and have been utilized for applications ranging from gaming (see [4] for review) to stroke rehabilitation [5]. However, there is a growing body of research into the development of a direct-speech BCI (DS-BCI), utilizing imagined speech as a communicative modality [6,7]. Imagined speech is the internal pronunciation of phonemes, words or sentences, independent of movement and without any audible output [7]. To date, it has been considered in relatively few BCI studies, as the field has favored MI, P300 or SSVEP paradigms (see [8] for review). However, a DS-BCI offers the possibility of a more naturalistic form of communication [9,10,11], relying on neural recordings corresponding to units of language rather than some unrelated brain activity [12,13]. One recent study has demonstrated the potential for spoken sentences to be synthesized from neural activity [14] and another has shown speech reconstruction directly from the auditory cortex while subjects listened to overt speech [15]. Non-invasive studies using magnetoencephalogram [16] and EEG [17] have demonstrated potential for decoding speech using these technologies. Several studies have used the advantage of non-invasive recording through EEG to investigate imagined speech as a communicative paradigm for BCIs (e.g., [17,18,19,20,21,22,23]). Traditional approaches to BCIs require feature extraction and classification algorithms designed to decode a specific control signal. EEG signals are non-linear and non-stationary and therefore highly complex [24], with classification typically achieved using features selected from some form of non-linear analysis and a machine learning (ML) algorithm. Most imagined speech EEG studies have used traditional BCI approaches to feature extraction and classification. Common spatial patterns (CSP) [25], autoregressive coefficients [26,27], spectro-temporal features [23], Riemannian manifold features [17] and Mel Frequency Cepstral Coefficients (MFCC) [18,28] are among those used to represent imagined speech EEG. As with feature extraction, imagined speech decoding has relied on several classification approaches typical to BCIs. The Support Vector Machine (SVM) has been the most common approach to date [18,20,23,25,28,29,30], showing results in the range of 77.5%–100% in binary vowel classification tasks [29] and 92.46% average accuracy in a two-class, meaning-based imagined speech task [20]. Linear Discriminant Analysis (LDA) has also been used for DS-BCI decoding [27,31], with one study reporting accuracies in the range of 66.4–76.0% in two-class imagined phoneme production tasks [31]. Other classifiers applied to imagined speech include Naïve Bayes [27,30], k-Nearest Neighbors [18,30], Random Forests (RdF) [30], Relevance Vector Machines [18], and Sparse Logistic Regression [22].

Deep learning (DL) techniques such as convolutional neural networks (CNN), recurrent neural networks (RNN) and others, have been successful in several fields of research, including computer vision [32] and automatic speech recognition (ASR) [33]. More recently, researchers have begun applying DL methods to BCI decoding challenges, and EEG analysis in general. One of the virtues of DL is the optimization of both feature extraction procedure and classifier in tandem, enabling a CNN for example, to learn both simultaneously [34]. DL has been applied across the spectrum of BCI and EEG paradigms, including MI [35] and epilepsy detection [24]. Of all DL methods, CNNs are the most common approach to EEG tasks, having been cited in 40% of studies [36]. Important works in the field include use of CNNs for SSVEP classification [37], P300 detection [38] and classification of mental workload from EEG using time-frequency transforms [39]. Recently, deep transfer learning with CNNs has been used for EEG-based BCI applications [40,41]. See [36] for a systematic review on EEG-based applications of DL.

Although a multilayer perceptron (MLP) ANN has been used to classify imagined speech with 63.2% mean accuracy in a yes vs. no task [21] and CNNs used to classify imagined speech in a multi-dataset study [42], there have been relatively few studies applying any form of DL in this area [43]. Indeed, it is still unclear whether DL methods provide consistent performance improvements over traditional ML approaches for EEG data [36,44]. Furthermore, despite recent studies implementing nested cross-validation (nCV) for hyperparameter (HP) optimization [45,46], robust consideration of HP selection in DL-EEG studies has been severely lacking in the literature, with almost 80% not mentioning HP searching at all [36]. Of the 21% of all DL EEG studies which considered HP optimization, the majority applied trial and error or grid search. Statistical analyses and effective reporting of results were almost completely absent from those studies that did implement some form of HP optimization. Sixteen studies have recently been highlighted by two topic-specific review papers for their use of HP optimization strategies [36,44]. However, further analysis of these studies indicates that it is almost impossible to infer any significant information on valid HPs for DL-EEG. Furthermore, there is virtually no comparison of the effects of different HP values, no discussion of the interaction between HPs and different models, and no statistical analysis of these effects.

Of the reviewed studies, nine considered CNNs in at least one form. Methods vary across these studies and include grid search [47,48], Bayesian methods [49,50,51] (one fails to report the specific approach [49]), trial and error [24,52], and unstated approaches, likely indicating trial and error [53,54]. In six of these studies, only partial results are reported in relation to HPs [24,48,50,51,53,54]. For example, in an otherwise excellent paper [50], only present optimal values for structural parameters were tested and the authors completely fail to report on the effects of optimizing learning rate and learning rate decay. There are similar omissions in relation to HP optimization in each of the six studies cited above. In the remaining three CNN studies highlighted by the review papers, no results on HP optimization are presented at all [47,49,52]. Not one of these studies presents any analysis of observed differences between HPs or their interaction with a given model, and none report any statistical analysis of results pertaining to HP optimization. In almost all of these papers, perhaps excluding [50,51], reporting of the approach to HP optimization is poor. Additionally, there are no interpretable results, no comparison of individual HPs or interaction between HPs, and no statistical analysis of anything relating to HPs. Other studies cited in the reviews [36,44], although employing DL models other than CNNs, follow this pattern of inadequate reporting of HP optimization.

It is the above points regarding the relative performance of DL and ML methods, and the lack of interest in HP optimization, that have informed the aims and methodology used here. There were three main aims to this study. First, we wanted to determine whether DL methods provided a significant improvement over benchmark ML approaches. Second, we wanted to undertake a robust, statistically validated, analysis of HP optimization, which would make it explicit which HPs were most effective. Third, we wished to investigate whether subject-specific HPs were always necessary, or whether generalization of HPs across subjects was feasible without significant loss of performance.

We take an EEG dataset recorded while participants perform imagined speech tasks in Spanish and perform classification using three different CNNs. The performances of the CNNs are compared to three traditional ML benchmark classifiers. The CNNs utilized have been selected for this study as they have each been specifically designed for EEG applications [34,55]. A nested approach to cross-validation, more commonly applied to standard ML problems, is here applied in a DL context. The nCV method facilitates HP optimization for all classifiers. Statistical analysis of the relative effects of the HP values on the CNNs’ performance, and the interactions between the HPs and the CNNs, provides important information for future approaches to decoding imagined speech, and EEG in general. Two modes of HP optimization are tested. The first is an intra-subject approach where subject-specific HPs are selected. The second is an inter-subject approach where a single set of HPs is selected for all subjects.

This paper presents the methodology, results and discussion of this study, in which we evaluate the relative performance of ML and DL approaches to imagined speech decoding, and the significance of HP optimization in relation to several different algorithms.

## 2. Methods

### 2.1. Dataset and Preprocessing Methods

**Figure 1:**
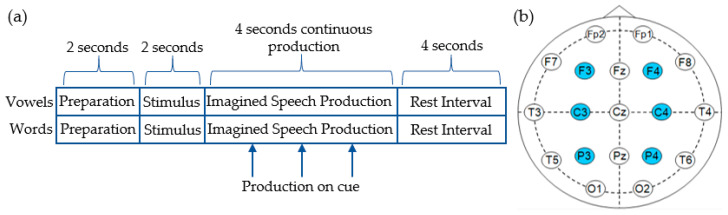
Experimental paradigm and montage used for data acquisition. (**a**) During vowel production, subjects performed imagined speech for the duration of the task period. During the words task, subjects received 3 audible cues instructing them to begin. (**b**) The 10–20 system of electrode placement was used, with the 6 electrodes (F3, F4, C3, C4, P3 and P4) highlighted.

The dataset used in this study was recorded by Pressel Coretto et al. [56] at the offices of the Laboratorio de Ingeniería en Rehabilitación e Investigaciones Neuromusculares y Sensoriales (LIRINS) in the Faculty of Engineering at the National University of Entre Ríos (UNER). EEG were obtained from 15 subjects while they performed overt and imagined speech production in response to two groups of prompts. The first group of prompts contained the vowels /a/, /e/, /i/, /o/ and /u/, with the second containing the six Spanish words “arriba”, “abajo”, “derecha”, “izquierda”, “adelante” and “atrás” (corresponding to the English words up, down, left, right, backward and forward). The vowel set was selected due to its acoustic stationarity and the lack of meaning associated with each unit. The words were selected as possible commands a user might use when interacting with a BCI. The subject cohort consisted of 15 healthy Argentinian college students (7 female), all native Spanish speakers, with a mean age of 25 years old. Subjects performed trials corresponding to each vowel and word 50 times (40 imagined, 10 overt). Each trial period of 4 s was preceded by a 2 s stimulus presentation period and followed by a 4 s resting interval (Figure 1a). Stimuli were presented in both visual and auditory form for all trials. During trials pertaining to vowels, subjects were asked to perform the task throughout the 4 s of the trial, while trials involving words were accompanied with three audible clicks indicating when participants should perform the task.

EEG signals were recorded using an 18-channel Grass^®^ analog amplifier model 8-18-36 and a Data Translation^®^ analog-to-digital converter board model DT9816, sampled at 1024 Hz. Six channels were used for data acquisition with electrodes positioned according to the 10–20 international system over F3, F4, C3, C4, P3 and P4 (Figure 1b). Recorded signals were filtered between 2 Hz and 40 Hz using a finite impulse response (FIR) bandpass filter and no further filtering was applied here. The filtered data were segmented in order to retain only signals recorded during imagined or audible speech production. For full details of the experimental protocol and recording technique, see [56]. Given the division of the imagined speech dataset two subcategories, and the fact that these signals were recorded using slightly different paradigms, a decision was taken to perform classification on both subsets.

Artefact detection and removal were implemented using Independent Components Analysis (ICA) with Hessian approximation preconditioning. EEG recordings were downsampled by a factor of 3 to improve computation time, and the FastICA method was selected for implementation. This approach required identification of an artefact, in this case an eye-blink artefact from subject one, which is then used as a template with which to measure other potential artefacts across subjects. All component maps (6 per subject) are mapped onto the template and those returning a correlation value above the threshold of 0.8 are identified as potential eye-blink artefacts. Visual inspection of components identified using this approach was performed before their final acceptance or rejection. In order to replicate the procedure in the original study [56], all data were downsampled to 128 Hz, resulting in 512 samples for each 4 s trial window. For the same reason, a sliding window was not applied.

### 2.2. Classification Techniques

Six classifiers were trained on the imagined speech EEG data. Three traditional machine learning approaches were used as benchmark methods, and three different CNN architectures used to determine the performance of deep learning approaches in comparison with standard machine learning methods.

#### 2.2.1. Benchmark Machine Learning Classifiers

In order to closely approximate the original study [56], the SVM and RdF classifiers were trained on relative wavelet energy (RWE) features extracted from the EEG. A discrete wavelet transform was first computed for each channel using five levels of decomposition. Fourth-order Daubechies were used to achieve the results presented in [56] and were therefore used here. A feature vector was constructed using the RWE of decomposition levels D2 (16–32 Hz), D3 (8–16 Hz), D4 (4–8 Hz), D5 (2–4 Hz), and A5 (<2 Hz), for each channel. This resulted in a 30-element feature vector for each trial. In the original study, the linear SVM implemented *one-vs-one* multi-class classification and multiple values for the *C* regularization parameter were evaluated. The RdF was constructed using different numbers of features (4, 5, 6) and trees (10, 50, 100, 200, 500). Three subjects were used to evaluate HPs and select the best configurations. Accuracies achieved in the original study [56] are presented in Section 3.5, alongside results from this study. Although the feature extraction methodology and the use of the SVM and RdF classifiers are retained from the original study, the approach to cross-validation and HP optimization is different. This is due to our focus on HP optimization and classification performance of the CNNs, and the requirement for application of a consistent methodology across classifiers. The methods implemented here are discussed in Section 2.3.

The rLDA is a regularized version of the LDA algorithm [57], which reduces the dispersion of eigenvalues of the sample covariance matrix when a diverging dimension p is large. As a classification method, it has been employed elsewhere in the decoding of EEG signals [57].

Filter bank common spatial patterns (FBCSP) is an approach to feature extraction widely used across multiple BCI paradigms [58]. The algorithm is used to calculate linear combinations of EEG channels which improve the discrimination of band power features between classes. The method can be broken down into four principle stages: a filter bank consisting of several bandpass filters (here 6), spatial filtering with the CSP algorithm, CSP feature selection and finally, classification of the selected features. The method has demonstrated impressive results in MI tasks, including being the winner in multiple EEG-decoding competitions (e.g., [59]). Its position as a leading method in BCIs, and the fact that FBCSP has been cited as an influence in the design of the CNNs being evaluated [34, 55], led to the selection of FBCSP as a reasonable benchmark. Here, six frequency bands are used to construct the filter bank. These are delta (2–4 Hz), theta (4–8 Hz), mu (8–12 Hz), lower beta (12–18 Hz), upper beta (18–28 Hz), and gamma (28–40 Hz). Three HPs were selected for optimization, i.e., (1). The number of spatial filters (nSF) used by the FBCSP algorithm, (2). The mutual information quantization level (MIQL), whereby mutual information is used to select or rank the best features, and (3). The number of features (NoF) used per trial. Further detail on these is presented in Section 2.3.

#### 2.2.2. CNN Architectures

**Figure 2:**
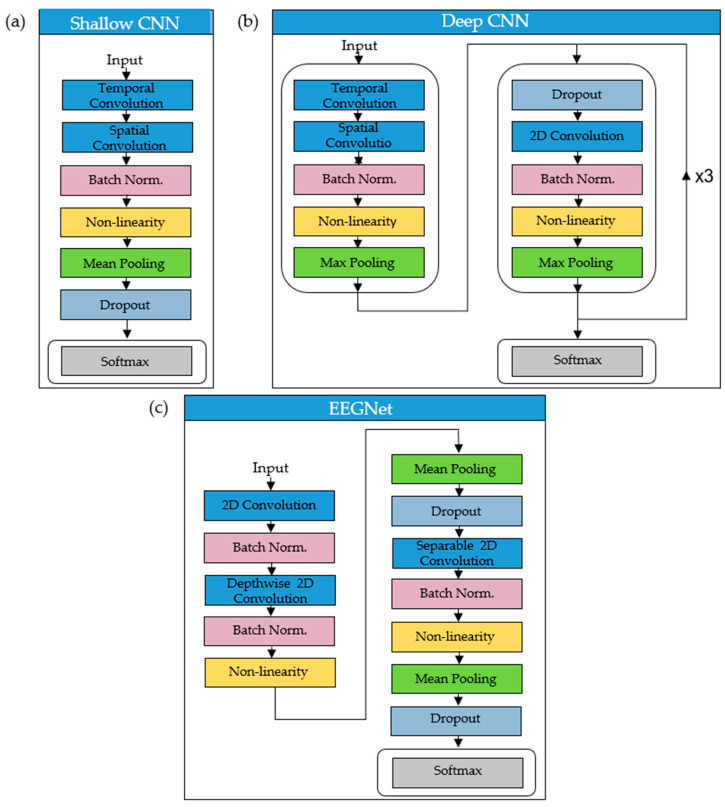
Network architectures of the shallow and deep CNNs, and the EEGNet. (**a**) is the shallow CNN: EEG signals are fed into the temporal convolution layer before proceeding through the spatial convolution layer. (**b**) is the deep CNN: it has the same initial structure as the shallow CNN, but with the addition of three identical convolution blocks. (**c**) is the EEGNet: EEG signals are fed into a 2D convolution layer. Depthwise and separable convolutions are also contained within its architecture.

The shallow CNN [34] is a network specifically designed to decode band power features from EEG. Constructed with a compact architecture (Figure 2a), the CNN has been conceived of for BCI applications with a feature extraction stage analogous to that of FBCSP [58]. Temporal and spatial convolutions performed in the first two layers of the shallow CNN correspond to transformations made during the bandpass and CSP spatial filtering stages of FBCSP. The output feature map is then fed through a squaring non-linearity, pooling layer (mean-pooling in [34]) and a logarithmic activation function to mimic the log-variance computation in FBCSP. Batch normalization [60] and dropout (0.5) [61] aid regularization (Figure 2a).

Presented in the same paper as the shallow CNN, the deep CNN was also designed for EEG decoding [34]. Based on similar networks used in computer vision (e.g., [32]), this CNN has been constructed to extract a wide range of features from EEG without being confined to specific feature types. The deep CNN has been shown to learn spectral amplitude changes in the input [62]. It has also demonstrated high sensitivity to phase features in the early stages of the network and amplitude features in later layers [62]. The deep CNN has been developed for the purpose of showing that generic CNN designs can be utilized as a decoding tool across multiple BCI/EEG paradigms [34]. Like the shallow CNN, the deep CNN has been applied to EEG relating to imagined or executed tasks [34], and normal and pathological EEG (where it marginally outperformed the shallow CNN) [63]. It has also been used for error decoding in an online BCI control paradigm [40], outperforming benchmark classifiers in this case. Additionally, the deep CNN has been used for decoding of intracranial recordings [64]. Figure 2b is a depiction of the architecture of the deep CNN. The first block consists of two convolutional layers, one to perform convolutional filtering over time and one to perform spatial filtering with weights for all possible pairs of electrodes. Following the first block are three identical convolutional blocks containing dropout (0.5) [61], convolution, batch normalization [60], exponential linear units (ELU) and max-pooling layers [34]. The output of the network is a softmax layer for classification. Full details of the shallow and deep CNNs are provided in the original paper [34].

EEGNet [55] is a CNN designed to be an effective cross-paradigm EEG classifier while, like the shallow CNN, retaining a compact architecture. EEGNet has been shown to generalize well across several BCI paradigms including sensorimotor rhythm (SMR) and movement-related cortical potential (MRCP) [55]. One of the guiding principles of the EEGNet composition is to facilitate training of CNNs on limited datasets, as is often the case in EEG studies. The design (Figure 2c) includes depthwise and separable convolutions acting as analogues to traditional EEG feature extraction techniques such as optimal spatial filtering and filter bank construction. The depthwise convolution is performed in the first block of the EEGNet, where a 2D convolutional layer is trained to output feature maps containing the EEG signals at different frequencies. Then the depthwise convolution is used to learn a frequency-specific spatial filter. Design of the initial layers of the EEGNet have been influenced by FBCSP [58]. In the second block of the network, separable convolution is applied, where a depthwise convolution is followed by a pointwise convolution. The virtue of this approach is its parameter reduction and decoupling of relationships across feature maps. Batch normalization [60], dropout (0.5) [61] and mean pooling aid regularization and dimensionality reduction. Non-linearity is executed in the form of ELU [65]. Finally, a softmax layer consisting of N units (N = number of classes), is used to classify features extracted by the convolutional layers. Full details of the EEGNet are provided in the original paper [55].

### 2.3. Method for Optimizing Hyperparameters

All classifiers were optimized using the nCV method, a typical approach to setting parameter values in traditional ML problems [66] but much less commonly used in DL optimization and HP validation [35]. It is often the case that studies implementing DL methods do not perform any cross-validation on training, or only perform a simple k-fold cross-validation scheme (e.g., [24]). While this is reasonable practice in fields such as computer vision, where enormous training sets are available, it is less so in BCIs, where smaller datasets increase the propensity for overfitted models. Therefore, a nCV approach has been employed here to facilitate HP optimization and a more reliable estimation of performance. Adapted from principles described in [66,67], the nCV method depicted in Algorithm 1 has been specifically tailored for DL validation. Firstly, the data are split into k folds, one of which is retained in the outer fold for testing. An inner fold is then instantiated with the remaining k-1 folds.

The combined inner-fold data are then re-split into k folds, and a validation accuracy is recorded for each HP combination. That is, for each set of parameters in the HP space, k values for model validation accuracy are returned. A mean validation accuracy is then calculated for each HP combination across all inner folds and the highest mean inner fold validation accuracy across folds is used to determine the optimum set of HPs. Concretely, a single set of HPs is selected for each classifier based on an aggregate of all inner-fold validation accuracies. The final model, with the optimized HPs, is then trained on outer-fold train folds and tested on the outer-fold test folds with the resulting classification accuracy used to report model performance. Model accuracy is reported as the mean test set accuracy across all k outer folds. In this study, 4-fold nCV has been implemented, resulting in each outer fold consisting of 75% training and 25% testing data. The training data are further split in *k* = 4 inner folds, resulting in 75% for training and 25% for validation.
**Algorithm 1**. Nested cross-validation (nCV)**Input:** Dataset *D* = ($x_{i}$, $y_{i}$), …,($\;x_{m}$, $y_{m}$) Set of hyperparameters *ϴ*
Classifier *C* Integer *k* **Outer fold:** **for each partition**
*D* into *D*_1_, *D*_2_,…,*D*_k_  **Inner fold:**
  Inner-fold data *iD* = **concatenate *iD*_1_,…,*iD_k−1_***
  **partition**
*iD* into i*D*_1_, i*D*_2_,…,i*D_k_*  **for**
*θ* in *ϴ*
    **for** i = 1….*k*      $Acc_{i,\theta }\;$= *C*(*iD_i_*, *θ*;$\;y_{i}$)      *Acc*(*θ*) = $\frac{1}{k}\overset{k}{\underset{i=1}{\sum }}Acc_{i,\theta }$ // mean accuracy for HP set     totalAcc(*θ*) = $\overset{k}{\underset{i=1}{\sum }}Acc\left(\theta \right)$ // mean HP accuracy                   for all inner folds θ* = argmax_θ_[totalAcc(*θ*)] // optimal set of hyper               parameters across all folds **for**
*i* = 1….*k*
$\;\;\;\;\;Acc_{i,\theta^{*}}$ = *C*(*D_i_*, θ^*^)
*Acc*(θ^*^) = $\frac{1}{k}\overset{k}{\underset{i=1}{\sum }}Acc_{i,\theta^{*}}$, $y_{i}$ // mean test accuracy                with a single set of HPs

Two modes of HP optimization are evaluated using the nCV approach discussed above. The first of these is an intra-subject approach in which HPs used to train the final model are selected on the basis of each individual participant’s inner-fold performance. Using this method, each classifier trained on a participant’s imagined word or vowel data was optimized with a bespoke set of HPs. The second mode is an inter-subject approach to nCV HP optimization. This technique uses the mean of validation accuracies across all inner folds aggregated across all subjects to select HPs for training a classifier. That is, a single set of HPs was used to train and test classifiers across all subjects.

Three SVM HPs were optimized using the nCV approach. The first of these was the kernel used by the SVM algorithm. As well as the linear kernel used in the original study [56], radial-basis function (rbf), polynomial and sigmoid kernels were tested. Four values for the regularization parameter, C, were also evaluated (0.1, 1, 10, 100), as were 4 values for the kernel coefficient known as gamma (g) (0.01, 0.1, 1, 10). Three different HPs were used to optimize the RdF. Just as in the original study [56], the NoF selected at each node and the number of trees used to construct the RdF were evaluated. In this study, NoF evaluated was 4, 5, 6 and 7, and the number of trees used to construct the RdF was 50, 100, 200 and 500. The third RdF HP evaluated was the minimum number of samples required at a leaf node (MSL) (1, 2, 3, 4). MSL is a smoothing parameter ensuring a split-point will only be considered if a minimum number of samples are present. As with the SVM and RdF, three different HPs were selected for optimization of the FBCSP-rLDA method. These were the nSF pairs (2, 3, 4, 5), MIQL (6, 8, 10, 12), and NoF per trial (8, 10, 12, 14).

Although HP optimization of the three benchmark approaches required evaluation of different HPs for each, one of the cornerstones of this study was to investigate the differential effects of HP optimization on the performance of the three different CNNs. Therefore, identical HPs were used to optimize each network. HPs selected for optimization of the CNNs were activation function, learning rate, number of training epochs and loss function. Four activation functions were evaluated with each CNN. The first of these was the ELU technique employed in the original papers presenting both the deep CNN [34] and the EEGNet [55]. The second is the squaring non-linearity implemented with the shallow CNN in the original paper [34] and also employed in [42]. The final two activation functions evaluated are the Rectified Linear Units (ReLU) and leaky ReLU techniques. Like ELU, ReLU adds non-linearity to the network and is a commonly used activation function for CNNs (see [68] for more information). A popular adaptation of the ReLU activation function, leaky ReLU assigns a non-zero slope to the negative part of the function and its features include adding sparsity to the network [24].

The second HP tested was the learning rate. The learning rate is a critical feature for training CNNs, and it was expected to have a significant impact on the performance of the three networks. A learning rate that is too low can lead to slow model convergence while a high learning rate can cause divergence [68]. Learning rates evaluated were 0.001, 0.01, 0.1 and 1.0. Epochs required for convergence of a CNN can vary depending on architecture, and this HP enabled analysis of whether the CNNs differed in this regard. The number of epochs used for training each of the CNNs was the third hyperparameter investigated. The number of epochs evaluated for training were 20, 40, 60 and 80.

Finally, two loss functions were evaluated by the HP search. Here, both negative log-likelihood (NLL) and cross-entropy (CE) are evaluated, as these were employed in the original studies. The NLL loss function is defined as:

(1) $$L\left(X,y\right)=\;-\overset{k}{\underset{i=1}{\sum }}y_{i}\log\left({\hat{y}}_{i}\right),$$

where y is the ground truth class label and $\hat{y}$ is the predicted output. CE is formulated as follows:

(2) $$L\left(X,y\right)=\;-\frac{1}{n}\overset{n}{\underset{i=1}{\sum }}\overset{l_{k}}{\underset{j=1}{\sum }}\left[y_{j}^{\left(i\right)}log{\hat{y}}_{j}^{\left(i\right)}+\left(1-y_{j}^{\left(i\right)}\right)\log\left(1-{\hat{y}}_{j}^{\left(i\right)}\right)\right].$$

In total, 128 different HP combinations are evaluated with each CNN architecture and each dataset.

### 2.4. CNN Training

Each CNN and dataset composition were trained on an intra- and inter-subject basis with a batch size of 64. Training each of the networks for classification requires the transformation of output values into conditional probabilities using the softmax function:

(3) $$p\left(l_{k}|f\left(X^{j};\theta \right)\right)=\;\frac{exp\left(f_{k}\left(X^{j};\theta \right)\right)}{\sum_{m=1}^{k}exp\left(f_{k}\left(X^{j};\theta \right)\right)},$$

where *X_j_* is the input, 𝑙_𝑘_ is a class label, *k* is the number of classes and *θ* are the parameters of the function. Probabilities assigned to class labels then facilitate computation of per example losses using a loss function. The loss function is minimized, and parameters are updated using an optimization algorithm with backpropagation. To preserve the consistency of the training procedure across the CNNs, a common optimization algorithm was selected. The ADAM optimizer [69] was used in both [31] and [57] and was therefore selected for this study. The braindecode repository was used for implementation [34] (https://github.com/TNTLFreiburg/braindecode). The number of trainable parameters for each CNN per dataset is provided in Supplementary Table S1. The compact EEGNet contains the fewest trainable parameters, followed by the shallow and then deep CNNs.

### 2.5. Statistical Analysis

The effect of HP values on inner-fold validation accuracy was evaluated to determine whether statistically significant differences existed between them. Given that each of the benchmark classifiers required different HPs to be optimized, a repeated-measures analysis of variance (ANOVA) was used to determine whether variation in HP values had any significant effect on accuracy. The use of a common set of HPs for optimization of the CNNs enabled use of a 2-way ANOVA HP × CNN type to determine whether the HPs had any significant effect on validation accuracy, and to test for effects of interaction between the HPs and the type of CNN.

The 2-way ANOVA CNN type × subject method was also used to determine whether final classification accuracies obtained by the six classification techniques exhibited statistically significant differences and to determine the significance of differences obtained when using the intra-subject approach and the inter-subject approach. In each of the above cases, when statistical significance was indicated, post-hoc statistical analysis was performed using a Tukey Honest Significant Difference (HSD) multiple comparisons test to account for differences between results [70].

## 3. Results

### 3.1. Hyperparameters and Kernels Selected for Benchmark Classifiers

Inner-fold validation accuracy was the metric used for selecting a given HP combination and the repeated-measures ANOVA used to determine whether any statistically significant differences existed between HP settings. With 15 subjects and 2 datasets (words and vowels), a total of 30 sets of HPs were selected for each classifier using the intra-subject method. Two sets of HPs were selected with the inter-subject approach.

For intra-subject HP optimization, the polynomial kernel was most often selected for training the optimum SVM (50%), while the linear kernel used in the original study was selected only twice. A *C* value of 100 was selected in 53.33% of cases when training the SVM. However, each *C* value was utilized multiple times. A g value of 1 was selected in 50% of cases and all values were selected multiple times. The effect of the kernel used for the SVM classifier was significant for the words data (F(3,59) = 2.967, *p* = 0.043) but not for the vowels (F(3,59) = 0.427, *p* = 0.735). Likewise, the effect of the *C* value was significant for words (F(3,59) = 3.213, *p* = 0.032) but was not for vowels data (F(3,59) = 0.066, *p* = 0.978). Differences observed due to varying the value for g were determined to be insignificant in either case (words showing a tendency towards significance: F(3,59) = 2.701, *p* = 0.058, vowels: F(3,59) = 0.101, *p* = 0.959). The Tukey post-hoc tests indicated significant differences only between the polynomial and sigmoid kernels (*p* < 0.05) and the *C* values 0.1 and 100 (*p* < 0.05). The HPs selected for training all subjects with the SVM were the polynomial kernel and a g value of 10 for both the words and vowels sets (Table 1). The data differed only in selection of *C* values (words: 10, vowels: 1).

The NoF for classification with the RdF was split between each of the four values evaluated (4 (26.67%), 5 (23.33%), 6 (23.33%), 7 (26.67%)). The number of trees used to construct the RdF was most often selected as 50 (36.67%), with 500 trees selected only 3 times in total. MSL was most often selected as 2. However, each of the four values was selected multiple times. None of the differences observed through variation in HPs for either words (NoF: F(3,59) = 2.243, *p* = 0.097; trees: F(3,59) = 0.310, *p* = 0.818; MSL: F(3,59) = 2.670, *p* = 0.060) or vowels (NoF: F(3,59) = 1.737, *p* = 0.174; trees: F(3,59) = 1.695, *p* = 0.183; MSL: F(3,59) = 2.361, *p* = 0.085) were determined to be significant, even though there is tendency towards significance with MSL. Inter-subject selection of HPs for the RdF was common across datasets for the number of trees (50) and MSL (2) (Table 1). The NoF used for classification differed between words (7) and vowels (5) data.

The nSF pairs selected for intra-subject optimization was most often 2 (40%). The value most commonly selected for MIQL intra-subjects was 4, although all four values were used multiple times (2 (16.67%), 4 (30%), 6 (26.67%), 8 (26.67%)). The NoF selected was most often 10 (36.67%). As with the RdF, none of the differences observed through variation of the HPs was determined to be significant for either words (nSF: F(3,59) =2.243, *p* = 0.097; MI: F(3,59) = 0.295, *p* = 0.829; NoF: F(3,59) = 0.123, *p* = 0.946) or vowels (nSF: F(3,59) = 0.85, *p* = 0.474; MI: F(3,59) = 0.09, *p* = 0.965; NoF: F(3,59) = 2.46, *p* = 0.076). Inter-subject selection of HPs for the rLDA/FBCSP were common across datasets for the NoF (10) but differed regarding nSF pairs and MIQL values (Table 1).

### 3.2. Hyperparameters Selected for CNNs

**Figure 3:**
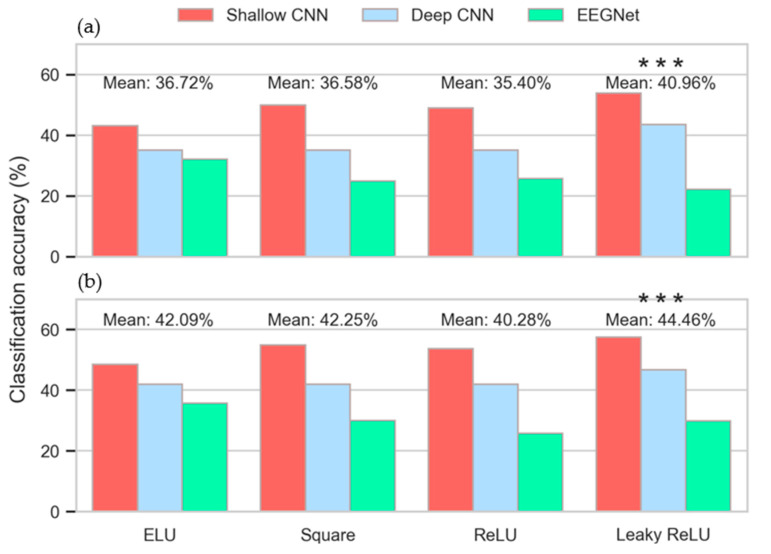
Mean inner-fold validation accuracy by activation function. (**a**) Imagined words (chance accuracy: 16.67%). (**b**) Imagined vowels (chance accuracy: 20%). *** *p* < 1 × 10^–6^.

The differential effects of the four activation functions evaluated are depicted in Figure 3. For both the words (Figure 3a) and vowels (Figure 3b) datasets, the leaky ReLU activation function achieved the highest mean inner-fold validation accuracies of 40.96% and 44.46% respectively. The 2-way ANOVA activation × CNN type indicated that the selection of different activation functions has a significant effect on performance relating to both words (F(3,14) = 30.03, *p* < 1 × 10^–12^) and vowels (F(3,14) = 45.46, *p* < 1 × 10^–16^. Furthermore, interaction between activation function and model was determined to be significant in both cases (words: F(6,14) = 77.64, *p* < 1 × 10^–31^, vowels: F(6,14) = 86.36, *p* < 1 × 10^–33^). The Tukey post-hoc tests determined that validation accuracies obtained with leaky ReLU were significant in comparison with those of the other three activation functions (words: *p* < 1×10^–8^, vowels: *p* < 1 × 10^–6^). The ReLU activation function achieved the lowest mean validation accuracies with both datasets. This result was significant in comparison with the scores obtained by the square and ELU functions for the vowels data (*p* < 1 × 10^–5^). Remaining differences were not significant.

**Figure 4:**
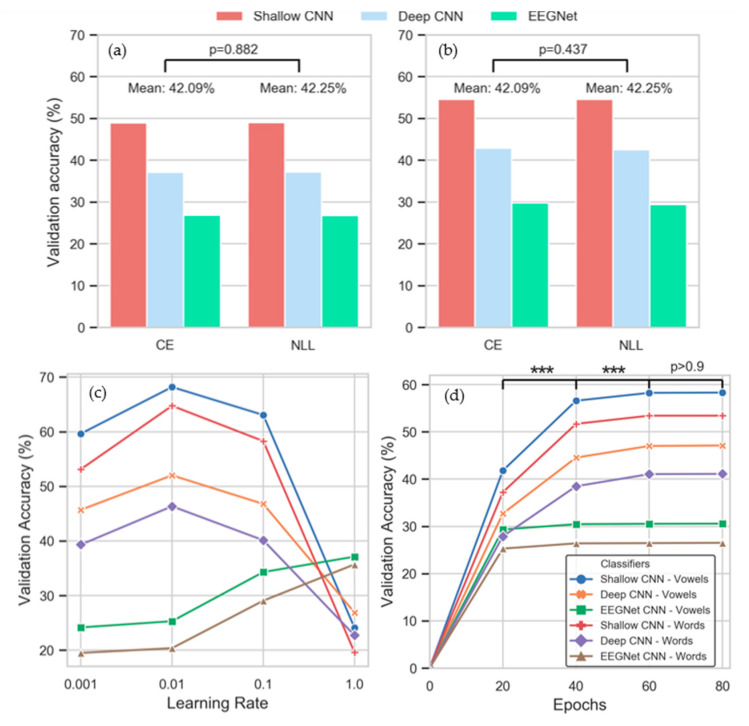
Inner-fold validation accuracy as a function of (**a**,**b**) loss (**c**) learning rate and (**d**) epochs. *** *p* < 1 × 10^–8^.

The 2-way ANOVA loss * CNN type determined that the effect of different loss functions was not significant for either words (F(1,14) = 0.022, *p* = 0.882) or vowels (F(1,14) = 0.62, *p* = 0.437) (Figure 4a,b). Similarly, the interaction between loss function and the type of CNN is not significant (words: F(2,14) = 0.02, *p* = 0.979, vowels: F(2,14) = 0.18, *p* = 0.837). Given these results, no post-hoc analysis was required.

Inner-fold validation accuracies obtained by the CNNs with different learning rates are plotted in Figure 4c. Here, the performances of the shallow and deep CNNs, and that of the EEGNet, diverge in relation to the effect of varying learning- rates. The shallow and deep CNNs both peak at 0.01, whereas the EEGNet’s mean accuracy increases with each step. The significance of the learning rate in inducing this divergence was confirmed by the 2-way ANOVA learning rate * CNN type with F(3,14) = 354.35, *p* < 1 × 10^–46^ for words and F(3,14) = 645.94, *p* < 1 × 10^–58^ for vowels. The interaction observed between the learning rate and the CNN architecture was highly-significant (words: F(6,14) = 329.32, *p* < 1 × 10^–55^, vowels: F(6,14) = 440.77, *p* < 1 × 10^–60^). The Tukey post-hoc tests determined that statistically significant differences exist between all possible combinations of learning rates examined, with *p* < 1 × 10^–8^ for all but 0.01 vs. 0.1 (*p* < 0.05, words; *p* = 0.80, vowels).

The effect of varying the number of epochs used for training the CNNs is shown in Figure 4d and Supplementary Table S2. As with the other HPs evaluated, the performance of the classifiers is consistent across the two imagined speech datasets, allowing for the number of classes per set. The validation accuracy of the EEGNet does not improve substantially above 20 epochs (vowels: 28.41% (20 epochs); 29.47% (80 epochs)). However, the performances of the shallow and deep CNNs continued to improve significantly up to 60 epochs, before beginning to level off. The effect of varying the number of epochs used for training the CNNs was found to be significant by the 2-way ANOVA Epochs * CNN type across both datasets (words: F(3,14) = 1457, *p* < 1 × 10^–71^, vowels: F(3,14) = 1471, *p* < 1×10^–71^). Furthermore, the interaction between the number of epochs used for training and the CNN architecture was determined to be significant for both words (F(6,14)= 293.97, *p* < 1 × 10^–53^) and vowels (F(6,14) = 295.10, *p* < 1 × 10^–53^). The Tukey post-hoc tests indicated that significant differences were present in all possible combinations of epochs between 20 and 60 (words: *p* < 1×10^–8^, vowels: *p* < 1 × 10^–8^). Differences observed between training across 60 epochs and 80 epochs were found to be insignificant for both words (*p* = 0.997) and vowels (*p* = 0.979).

### 3.3. Intra-Subject Selection of Hyperparameters

**Figure 5:**
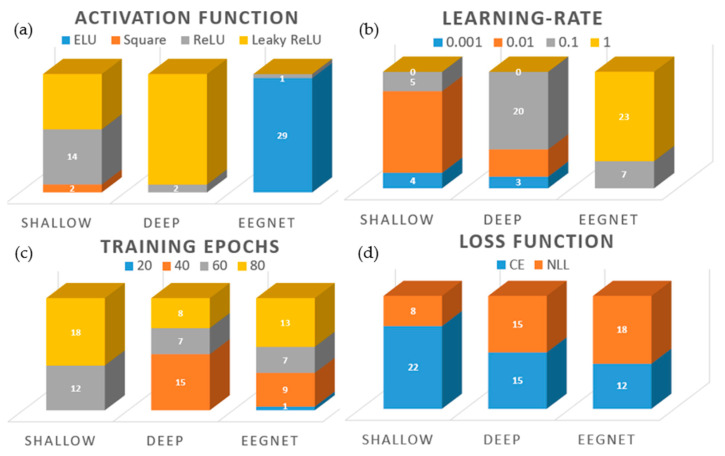
Distribution of hyperparameters selected for different CNN architectures. (**a**) Number of instances each activation function was selected. (**b**) Number of instances each learning rate was selected. (**c**) Number of instances each number of training epochs was selected. (**d**) Number of instances each loss function was selected.

The distribution of HPs selected using the intra-subject method is presented in Figure 5. ReLU and leaky ReLU were selected most often as the activation function for intra-subject training of the Shallow CNN, and leaky ReLU is selected in 28 out 30 cases for training the deep CNN (Figure 5a). In total contrast, the EEGNet selects the ELU activation in 96.67% of cases. The square activation function is selected only twice (shallow CNN). The selection of an optimum learning rate is considerably different across the three CNNs (Figure 5b). The shallow CNN selected 0.01 most often, whereas the deep CNN selects 0.1 in 66.67% of the cases. However, both of these networks make use of all learning rates below 1. Once again, the EEGNet differs significantly from the other CNNs. With regards to learning rate, the EEGNet selects a value of 1 in 76.67% of cases. There is less variation between the CNNs in relation to the number of training epochs required (Figure 5c). In particular, the deep CNN and EEGNet exhibit similar distributions of 40, 60 and 80 epochs for training. However, the EEGNet is the only one of the three to select 20 as the optimal number of epochs. The shallow CNN trends towards a greater number of training epochs, either 60 or 80 in all cases. Statistical analysis presented in the previous section indicated that differences obtained when training with the CE or NLL loss functions were not significant. Further evidence of this is apparent in the distribution of loss functions selected for intra-subject optimization (Figure 5d). Although the shallow CNN and the EEGNet do slightly favor one loss function over the other, the deep CNN does not (50% each).

### 3.4. Inter-Subject Selection of Hyperparameters

HPs selected using the inter-subject method are presented in Table 2. The shallow and deep CNNs’ selection of HPs were almost identical to each other, with activation function (leaky ReLU), learning rate (0.1) and number of training epochs (60) the same across data types. The only anomaly was the selection of NLL as the loss function for the shallow CNN when training on imagined words. However, as the results of the 2-way ANOVA indicated that there were insignificant differences between the two loss functions, this is not a critical variation. Although higher mean inner-fold validation accuracies for the shallow and deep CNNs were achieved with a learning rate of 0.01 (Figure 4c), when the best HP combination was selected, the learning rate was 0.1. Just as with the intra-subject optimization, HPs selected for the EEGNet differed considerably from the other CNNs. Of these, the selection of ELU as the activation function and 1 as the learning rate are the most important. We have already seen that differences between training on 60 or 80 epochs were not significant (*p* > 0.9) and that the selection of loss function is not critical. The selection of ELU and a learning rate of 1 is expected given the results presented in Figure 3, Figure 4c and Figure 5a,b. Although leaky ReLU returned higher mean inner-fold accuracies, the EEGNet performed best with ELU. The reason for this selection is the significant effect of the higher learning rate used here by the EEGNet and the impact this has on the activation functions.

### 3.5. Classification Performance

**Figure 6:**
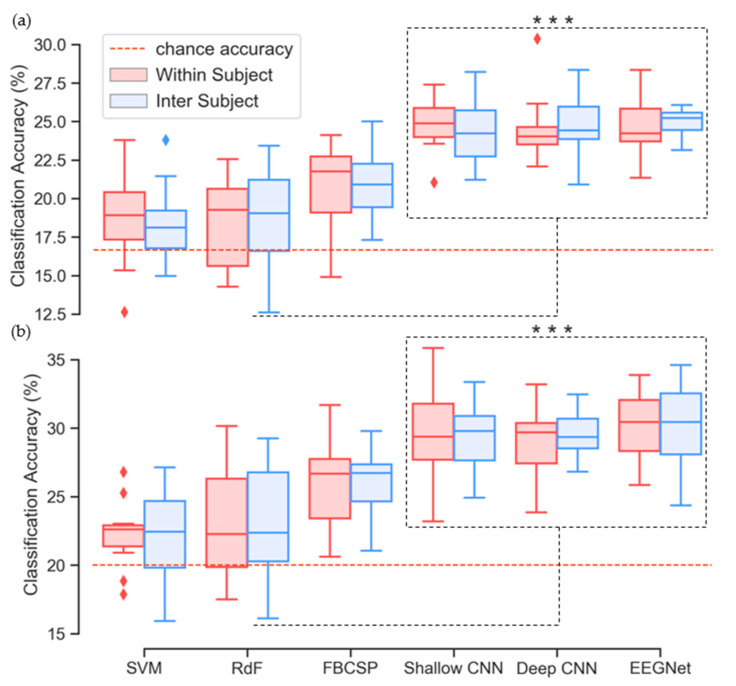
Classification accuracies for (**a**) imagined words and (**b**) imagined vowels using intra- and inter-subject modes. *** *p* < 1 × 10^–7^.

Classification accuracies for both intra-subject and inter-subject approaches are presented in Figure 6 and Table 3 (words) and Table 4 (vowels). Corresponding precision scores (true positives/true positives + false positives) are presented in Supplementary Tables S3 and S4. The 2-way ANOVA determined that results obtained by using different classification methods were significant for both words (F(5,14) = 121.32, *p* < 1 × 10^–32^) and vowels (F(5,14) = 77.596, *p* < 1 × 10^–26^). However, the effects of using an intra- or inter-subject method for selecting HPs were not (words: F(1,14) = 0.151, *p* = 0.699; vowels: F(1,14) = 0.626, *p* = 0.432). Results obtained using the SVM and RdF classifiers trained on RWE features were comparable with those presented in the original paper (words: SVM: 18.26%, RdF: 18.58%; vowels: VM: 21.94%, RdF: 22.32%) [60]. The SVM achieved accuracies of 18.71% (intra) and 18.36% (inter) with the imagined words data and the RdF achieved comparable performances of 18.37% (intra) and 18.72% (inter) with the same data. Results from the vowel dataset also showed similar performance between our benchmark and previous results. Here, we obtained accuracies of 22.23% (intra) and 22.25% (inter) with the SVM and 23.08% (intra) and 23.23% (inter) with the RdF. For both imagined words and imagined vowels, the Tukey post-hoc tests determined that the different results obtained by the SVM and RdF approaches were not significant. The FBCSP-trained rLDA achieved higher classification accuracies than both the SVM and RdF for both imagined words (intra: 20.77%, inter: 21.03%) and vowels (intra: 25.82%, inter: 26.22%). The improvement in performance associated with the FBCSP approach ranges from 2.05% to 2.67% for words and 2.59% to 3.99% for vowels. The post-hoc tests also revealed that the FBCSP-trained rLDA method performed significantly better that the original benchmark approaches for both data types (*p* < 1 × 10^–6^, *p* < 1 × 10^–5^). For imagined words (chance: 16.67%), each of the CNNs achieved higher mean accuracies for both intra- and inter-subject optimization than the three benchmark methods (Table 3)). The Tukey post-hoc tests confirmed that the superior accuracies of the CNNs in comparison with the benchmark approaches were significant in relation to imagined words (*p* < 1 × 10^–6^). The highest mean accuracy was recorded by the EEGNet (24.90%, std. 1.27%). However, individual differences among the three CNNs were not significant.

The three CNNs also outperformed the benchmark approaches on the imagined vowels task (Table 4). All three CNNs obtained mean accuracies ranging from 28.95% to 30.25% for both intra- and inter-subject optimization. Once again, the performance of the CNNs in comparison with those of the benchmark methods was determined to be significant by the Tukey post-hoc tests (*p* < 1 × 10^–6^). However, individual differences among the three CNNs were also not significant when trained on imagined vowels data.

## 4. Discussion

The study presented here is one investigating the effects of varying HPs used for constructing and training multiple CNNs, and the impact of optimizing HPs using intra- and inter- subject methodologies. Furthermore, overall classification accuracies of imagined words and vowels obtained using CNNs were compared to three traditional ML approaches. Results obtained from the nCV approach to HP optimization indicate the importance of HP selection when implementing CNNs. For all but the loss function, varying the values of HPs resulted in effects that were both significant across the different HP values and the different CNN architectures. Unsurprisingly, given its current prominence as a non-linear activation function for CNNs, leaky ReLU achieved highest mean validation accuracy scores (Figure 3). This result was reflected in selection of leaky ReLU for most intra-subject cases and all inter-subject cases for the shallow and deep CNNs. However, ELU was the optimal activation function for use with the EEGNet.

Smaller learning rates (0.001–0.1) achieved the best results with both the shallow and deep CNNs but a swift drop off was apparent when the learning rate was increased to 1. In contrast, the EEGNet performed best with a learning rate of 1, although it is unclear whether this trend would continue if the learning rate was further increased. While accuracies obtained by the EEGNet only increase in small increments after 20 epochs (indicating that less training is required for this network), it nevertheless selected 80 epochs for inter-subject training. In contrast, the shallow and deep CNNs take statistically significant leaps when trained on 40 (*p* < 1 × 10^–8^) and 60 epochs (*p* < 1 × 10^–60^), respectively. This is reflected in the results of inter-subject HP optimization, where the number of epochs selected is consistently 60 (Table 2).

Taken together, these results highlight the importance of selecting reasonable HPs, and the selection of learning rates and activation functions indicate that these two HPs are critical to performance (Table 2). However, they also indicate how critical the interaction among HPs is, and how critical the interaction between HPs and the network architecture is. These interactions are not always considered when a CNN is being implemented for classification tasks [36]. The importance of interactions between HPs within a given network is evidenced by the selection of a learning rate for the shallow and deep CNNs. A learning rate of 0.001 clearly obtains the highest inner-fold validation accuracy when evaluated independently of the other HPs (Figure 4c). However, as selection of HPs for final model training is based on interaction between different HP combinations, a different learning rate (0.1) is selected as the optimal (Table 2). The reason for this apparently incongruous result is that the leaky ReLU activation function performed better with a learning rate of 0.1 than 0.01, whereas the learning rate of 0.01 performed best across all activation functions. That is, the HPs selected (Table 2) are based on validation accuracy as a function of the set (activation function, learning rate, loss and number of epochs), rather than individual maximum values. The criticality of interactions between the HPs and the specific CNNs is indicated by the differential effects of activation functions, learning rates and number of epochs on the three CNNs. The dramatic differences obtained between the shallow and deep CNNs, and the EEGNet, are particularly visible in Figure 4c,d, where the EEGNet’s optimal learning rate is 1 and it is able to achieve similar accuracies after 20 epochs and 80 epochs. It is also clear from Figure 5 and Table 2 that the different CNNs respond differently to the various HPs tested.

Results in Section 5 indicate that optimization of HPs evaluated for the SVM, RdF and rLDA resulted in differences that were either not significant at all or only significant with 0.01 < *p* < 0.05. In comparison, HPs tested with the CNNs were all highly significant, with the exception of the loss functions. These results suggest that, although time-intensive, HP optimization is of greater importance with regards to CNN approaches than it is to traditional ML methods.

In this work, we have detailed the HPs that worked best for each of the CNNs when training on imagined speech EEG data (Table 2, Figure 5). We have also presented future users of these networks with effective HPs in relation to activation function, learning rate and number of training epochs. Along with previous works [34,42,55], this should provide researchers with a reasonable benchmark from which to approach other classification problems with these CNNs. The nCV technique applied here is one not often applied in DL contexts [36], but due to the relatively small quantity of training data available, it was necessary to use this method to enhance model robustness.

Use of an inter-subject or intra-subject method for selecting HPs was not significant for either words (F(1,14) = 0.151, *p* = 0.699) or vowels (F(1,14) = 0.626, *p* = 0.432)). Although HP optimization has clearly been shown to be a significant factor in the performance of the CNNs, it is not necessarily important for these HPs to be optimized on an inter-subject basis. High inter-subject variability has been a limiting-factor for EEG applications [36], so the fact that final accuracies obtained could be achieved with global selection of HPs could have important consequences for inter-subject training.

In each case, the CNN architectures achieved higher classification accuracies than the benchmark classifiers and these results were significant (*p* < 1 × 10^–7^). The range of improvement that the CNNs achieved in comparison with the benchmark (words: 3.94–6.54%; vowels: 3.78–8.02%) is consistent with the reported 5.4% median improvement attributed to DL approaches in comparison with traditional ML methods for EEG classification [36]. The range of accuracies achieved by the final models (words: 24.35–24.90%; vowels: 28.95–30.25%) indicate that the different CNN architectures are similarly capable of decoding imagined speech EEG. This is despite the significant differences in the selection of HPs required to optimize the different networks, and different inner-fold performance on the validation set. It should also be noted that the CNNs have vastly different numbers of trainable parameters (Supplementary Table S1), with the compact EEGNet much less computationally complex than either the shallow or deep CNNs; an important factor in interpreting overall performance. In fact, complexity is a key aspect in determining the overall efficacy of a model as it comes with a cost in terms of training time required. Due to the large number of trainable parameters in CNNs, traditional machine learning approaches have been shown to be less time-consuming [71]. However, this work has demonstrated that CNNs provide a statistically significant advance on traditional approaches. This, and the fact that utilizing trained CNNs to make predictions (with GPUs to help for fast inference) does not differ greatly from traditional methods, supports the use of CNNs for imagined speech decoding. Taken together, the results support the claims of the authors’ of the original papers that these CNNs can generalize well across BCI paradigms [34,55] and indicate the potential of DL methods for decoding imagined speech EEG.

Regarding the classification results, it must be made clear that the observed performances of the models are not at a level that would be required for a working DS-BCI. For such an important mode of communication it is imperative that users be supplied with a highly accurate and robust system. Despite recent advances [10,14] and high levels of interest, the field is not yet at that stage. Several avenues for investigation are available to researchers seeking to make progress in this regard. These include systematic improvements such as increasing the number of trials and the number of recording channels used. Additionally, experimental improvements can be sought through investigation of the neurolinguistic properties of parts-of-speech and the impact of stimulus presentation methods. However, the findings presented here compare favorably with other studies in the field. Many previous studies have applied binary classification paradigms to the study of imagined words, with average accuracies of 58% [18], 80.05% [17] and 92.46% [20] demonstrating the potential for decoding imagined speech EEG. Mean accuracy of 50.06% in a 3-class classification task involving short imagined words has been reported [17]. In this work, we have demonstrated the potential to decode a greater range of words from imagined speech EEG by classifying six words with a mean accuracy of 24.90% (EEGNet) and highest single-subject mean of 30.36% (subject 13, deep CNN).

Imagined vowel classification has previously been shown to be feasible, with results in the range of 48.96% for 3-class [17], and 77.5–100% for binary classifiers [29]. Here, we have shown that classification of multiple imagined vowels is possible with a mean accuracy of 30.25% (chance: 20%) and highest single-subject mean of 35.83% (subject 7, shallow CNN), providing evidence that linguistic units below the level of the word or sentence, i.e., phonemes, can be distinguished from EEG. However, the promise indicated by these results must be tempered by acknowledgement of the limitations of the study. First among these is the size of the imagined speech dataset and the relatively small number of trials per class (maximum: 40 [56]). The CNNs were reasonably robust given the number of training samples afforded by the dataset. Although the test accuracy tended to be lower than the training accuracy, possibly indicating some overfitting, this is unsurprising given the small sample size of data which makes it difficult for CNNs to generalize. It is well known that more data typically improves the performance of a CNN, and we agree with the recommendations in [71], where a search for increasing sample sizes is advocated as a method to ameliorating the large variance and errors associated with cross-validation approaches and small datasets. The second limitation is the small number of EEG channels used in data acquisition. Six electrodes is a small number and restricts the spatial resolution of the EEG data. Given that CNNs work to capture spatial dependencies in the data, the shortage of electrodes likely impacted negatively on overall performance. Other studies related to imagined speech decoding have typically used montages ranging from 16 [30] to 64 [17] electrodes, thus providing greater information to decoding models. If a DS-BCI is to become a functional technology, full consideration of each of these issues is essential. It must also be noted that any additional complexity emerging from the use of deeper neural network architectures and potentially longer training times, must be accounted for in the development of real-time BCIs using these models. It can be observed in Figure 5c that optimizing for accuracy often resulted in selection of 80 training epochs. However, the additional training time required may make the total benefit of this optimization negligible in a real-time scenario.

A weakness of the present study is that the experimental paradigm does not facilitate any analysis of the differential effects of the different stimuli as the prompts are presented concurrently. Additionally, the paradigm has not accounted for possible effects of user training on BCI performance. Subject training, in the form of task repetition and learning through neurofeedback (e.g., [72]), has been shown to improve the performance of BCIs using other paradigms. It is likely that similar improvements to DS-BCI performance can be obtained through use of effective training strategies aimed at enhancing a user’s ability to interact with a given system. Although the majority of DS-BCI studies have relied on single-session protocols [23,56], others have used multiple sessions for data collection [17,21]. Studies have demonstrated classification of imagined speech EEG with as few as 30, 40 or 50 trials per class [25,56]. However, higher-volume data are required to fully exploit DL models such as CNNs. Multi-session protocols offer several advantages including user training, collection of a greater number of trials per class, and analysis of multiple paradigms. For example, using up to three recording sessions, [17] were able to collect 100 trials per class while also investigating effects of using vowels, short words and long words. Use of multi-session training and neurofeedback within future experimental protocols is required to ascertain the impact of these strategies.

## 5. Conclusions

In this study, we evaluated the performance of three different EEG-decoding CNN architectures on imagined speech EEG. Imagined words and vowels were each used to train the CNNs, as well as benchmark SVM, RdF and rLDA classifiers. Each of the classifiers was optimized using a nCV approach to HP optimization. Both an intra- and an inter-subject method of HP selection were implemented and statistically evaluated.

The results indicated that effective selection of HPs when using these CNNs for EEG decoding is critical to performance. In particular, the activation function, learning rate and number of training epochs were determined to have a highly significant impact on the model. Interaction between the different HPs was found to be significant, so that selection of an optimal HP value was dependent upon the values of other HP values. Additionally, the interaction between the HPs investigated and the different CNNs evaluated was also significant. Similarities between the shallow and deep CNN resulted in very similar selection of HPs. In contrast, the EEGNet selected a very different set of HPs for optimal performance.

The results demonstrate that all three CNN networks outperform the benchmark classifiers in terms of classification accuracy, and that these results are statistically significant. However, variation in the classification performance of the three CNNs was small and not statistically significant. The effects of optimizing the classifiers using intra- and inter-subject methods was not significant. The use of nCV within the context of DL provides a way for improving the robustness of the training process with relatively small datasets. This study advances the field of DS-BCIs by demonstrating the effectiveness of DL approaches in decoding imagined speech EEG and by demonstrating the highly significant effect of HP optimization.

## Figures and Tables

**Table 1:** Selected inter-subject hyperparameters (baselines).

	SVM			RdF			FBCSP		
	Kernel	*C*	*g*	NoF	Trees	MSL	nSF	MIQL	NoF
Words	poly	10	10	7	50	2	2	8	10
Vowels	poly	1	10	5	50	2	4	4	10

**Table 2:** Selected inter-subject hyperparameters (CNNs).

	Shallow CNN		Deep CNN		EEGNet	
	Words	Vowels	Words	Vowels	Words	Vowels
Activation Function	leaky ReLU	leaky ReLU	leaky ReLU	leaky ReLU	ELU	ELU
Learning Rate	0.1	0.1	0.1	0.1	1	1
Epochs	60	60	60	60	80	80
Loss	CE	NLL	CE	CE	NLL	NLL

**Table 3:** Average accuracies for the benchmark and CNN classifiers trained on imagined words.

	Benchmark Methods						CNN Methods					
	SVM		RdF		rLDA		Shallow		Deep		EEGNet	
	Intra	Inter	Intra	Inter	Intra	Inter	Intra	Inter	Intra	Inter	Intra	Inter
Accuracy	18.71	18.36	18.37	18.72	20.77	21.03	24.88	24.35	24.42	24.78	24.46	24.90
Std.	2.90	2.46	2.83	3.16	2.66	2.18	1.59	1.95	1.91	1.78	1.75	0.93
Max.	23.79	23.79	22.56	23.42	24.13	25.00	27.38	28.22	30.36	28.36	28.35	26.54

**Table 4:** Average accuracies for the benchmark and CNN classifiers trained on imagined vowels.

	Benchmark Methods						CNN Methods					
	SVM		RdF		rLDA		Shallow		Deep		EEGNet	
	Intra	Inter	Intra	Inter	Intra	Inter	Intra	Inter	Intra	Inter	Intra	Inter
Accuracy	22.23	22.25	23.08	23.23	25.82	26.22	29.62	29.39	29.06	29.58	30.08	30.25
Std.	2.968	3.33	3.88	4.13	3.13	2.32	3.45	2.51	2.58	1.75	2.67	2.73
Max.	26.78	27.12	30.16	29.25	31.68	29.77	35.83	33.36	33.20	32.46	32.38	35.18

## Supplementary Materials

The following are available online at https://www.mdpi.com/1424-8220/20/16/4629/s1, Table S1: Number of trainable parameters per CNN per dataset; Table S2: Inner-fold validation accuracies for number of epochs; Table S3: Average precision scores for the benchmark and CNN classifiers trained on imagined words; Table S4: Average precision scores for the benchmark and CNN classifiers trained on imagined vowels.
