# Supplementary material for: Evaluation of Hyperparameter Optimization in Machine and Deep Learning Methods for Decoding Imagined Speech EEG
Source: Sensors (Basel). 2020 Aug 17;20(16):4629. doi: 10.3390/s20164629 (PMC7472624; doi:10.3390/s20164629)
Supplement: Supplementary file 1 [file sensors-20-04629-s001.pdf]

sensors

*Article*

# Evaluation of Hyperparameter Optimization in Machine and Deep Learning Methods for Decoding Imagined Speech EEG

**Ciaran Cooney [1,*], Attila Korik [1], Raffaella Folli [2] and Damien Coyle [1]**

[1] Intelligent Systems Research Centre, Ulster University, Londonderry BT48 7JL, UK; korik-a@ulster.ac.uk (A.K.); dh.coyle@ulster.ac.uk (D.C.)
[2] Institute for Research in Social Sciences, Ulster University, Jordanstown BT37 0QB, UK; r.folli@ulster.ac.uk
* Correspondence: cooney-c@ulster.ac.uk

Received: 10 June 2020; Accepted: 13 August 2020; Published: date

## Supplementary

**Table 1 S1.** Number of trainable parameters per CNN per dataset.

| | Shallow CNN | | Deep CNN | | EEGNet | |
|---|---|---|---|---|---|---|
| | Words | Vowels | Words | Vowels | Words | Vowels |
| Trainable parameters | 49562 | 48245 | 268481 | 268260 | 5990 | 5477 |

**Table 2 S2.** Inner-fold validation accuracies for number of epochs.

| | Shallow CNN | | Deep CNN | | EEGNet | |
|---|---|---|---|---|---|---|
| | Words | Vowels | Words | Vowels | Words | Vowels |
| 20 | 37.22 | 41.80 | 27.88 | 32.70 | 25.29 | 29.33 |
| 40 | 51.69 | 56.59 | 38.49 | 44.55 | 26.40 | 30.46 |
| 60 | 53.43 | 58.26 | 41.06 | 47.01 | 26.45 | 30.54 |
| 80 | 53.43 | 58.31 | 41.10 | 47.12 | 26.51 | 30.57 |

**Table S3.** Average precision scores for the benchmark and CNN classifiers trained on imagined words.

| | Benchmark Methods | | | | | | CNN Methods | | | | | |
|---|---|---|---|---|---|---|---|---|---|---|---|---|
| | SVM | | RdF | | rLDA | | Shallow | | Deep | | EEGNet | |
| | Intra | Inter | Intra | Inter | Intra | Inter | Intra | Inter | Intra | Inter | Intra | Inter |
| Accuracy | 18.85 | 18.42 | 18.33 | 18.70 | 20.85 | 20.97 | 24.91 | 24.29 | 24.45 | 24.86 | 24.46 | 24.88 |
| Std. | 2.85 | 2.44 | 2.82 | 3.17 | 2.68 | 2.14 | 1.56 | 1.95 | 1.89 | 1.79 | 1.72 | 0.92 |
| Max. | 23.81 | 23.76 | 22.61 | 23.40 | 24.10 | 25.04 | 27.39 | 28.26 | 30.30 | 28.32 | 28.35 | 26.51 |

**Table S4.** Average precision scores for the benchmark and CNN classifiers trained on imagined vowels.

| | Benchmark Methods | | | | | | CNN Methods | | | | | |
|---|---|---|---|---|---|---|---|---|---|---|---|---|
| | SVM | | RdF | | rLDA | | Shallow | | Deep | | EEGNet | |
| | Intra | Inter | Intra | Inter | Intra | Inter | Intra | Inter | Intra | Inter | Intra | Inter |
| Accuracy | 22.20 | 22.26 | 23.13 | 23.25 | 25.75 | 26.16 | 29.67 | 29.37 | 29.09 | 29.62 | 30.04 | 30.19 |
| Std. | 2.97 | 3.35 | 3.89 | 4.10 | 3.11 | 2.33 | 3.42 | 2.51 | 2.54 | 1.77 | 2.67 | 2.71 |
| Max. | 26.76 | 27.15 | 30.13 | 29.24 | 31.71 | 29.73 | 35.85 | 33.37 | 33.24 | 32.47 | 32.31 | 35.15 |
